# Personalised exercise therapy and self-management support for people with multimorbidity: feasibility of the MOBILIZE intervention

**DOI:** 10.1186/s40814-023-01242-0

**Published:** 2023-01-18

**Authors:** Søren T. Skou, Rasmus H. Brødsgaard, Mette Nyberg, Mette Dideriksen, Uffe Bodtger, Alessio Bricca, Madalina Jäger

**Affiliations:** 1 Research Unit for Musculoskeletal Function and Physiotherapy, Department of Sports Science and Clinical Biomechanics, University of Southern Denmark, Campusvej 55, 5230 Odense M, Denmark; 2 The Research Unit PROgrez, Department of Physiotherapy and Occupational Therapy, Næstved-Slagelse-Ringsted Hospitals, Region Zealand, 4200 Slagelse, Denmark; 3 Pulmonary Research Unit Region Zealand (PLUZ), Department of Respiratory Medicine, Zealand University Hospital Naestved, 4700 Naestved, Denmark; 4 Institute for Regional Health Research, University of Southern Denmark, 5230 Odense M, Denmark

**Keywords:** Multimorbidity, Exercise, Self-management, Rehabilitation, Quality of Life, Physical Function, Feasibility

## Abstract

**Background:**

Exercise therapy is safe and effective in people with single conditions, but the feasibility in people with two or more conditions is unclear. Therefore, the aim was to evaluate the feasibility of exercise therapy and self-management in people with multimorbidity prior to a randomised, controlled trial (RCT).

**Methods:**

This was a mixed-methods feasibility study performed in two general hospitals and one psychiatric hospital. 20 adult patients (8 females; mean age (SD) 67 (6.9)) with at least two long-term conditions and a score of ≥ 3 on Disease Burden Impact Scale for at least one condition (at least moderate limitations of daily activities) and of ≥ 2 for at least one other condition. Patients with unstable health conditions, at risk of serious adverse events (SAE) or with terminal conditions were excluded. Participants received 12 weeks of exercise (18 60-min group-based and 6 home-based sessions) and self-management support (6 90-min group-based sessions) supervised by physiotherapists. Pre-defined progression to RCT criteria were the primary outcomes and included recruitment rate (acceptable 20 participants in 3 months), retention through follow-up (75% retention), compliance (75% complete > 9 of exercise and > 3 self-management sessions), outcome burden (80% do not find outcomes too burdensome), improvement in quality of life (EQ-5D-5L) and function (6-min walk test; ≥ 50% experience clinically relevant improvements) and intervention-related SAEs (No SAEs). Furthermore, a purposeful sample including eleven participants and two facilitators were interviewed about their experiences of participating/facilitating. Qualitative data was analysed using thematic analysis.

**Results:**

Recruitment rate (20 in 49 days), retention (85%), outcome burden (95%), and SAEs (0 related to intervention) were acceptable, while compliance (70%) and improvements (35% in quality of life, 46% in function) were not (amendment needed before proceeding to RCT). The intervention was found acceptable by both participants and physiotherapists with some barriers among participants relating to managing multiple chronic conditions while caring for others or maintaining a job. Physiotherapists expressed a need for additional training.

**Conclusions:**

Exercise therapy and self-management are feasible in people with multimorbidity. The subsequent RCT, amending the intervention according to progression criteria and feedback, will determine whether the intervention is superior to usual care alone.

**Trial registration:**

ClinicalTrials.gov registration: NCT04645732

Open Science Framework https://osf.io/qk6yg/

**Supplementary Information:**

The online version contains supplementary material available at 10.1186/s40814-023-01242-0.

## Key messages regarding feasibility

- What uncertainties existed regarding the feasibility?
  While the feasibility and effects of exercise therapy and self-management support in people with single conditions had been investigated, it was unclear if such interventions were also feasible in people with multimorbidity (two or more conditions in the same person). This is important, as people with multimorbidity have poorer physical and mental health and are at higher risk of serious adverse events than people with single conditions and often require a patient-centred approach to care as opposed to the disease-centred approach applied in people with single conditions.
- What are the key feasibility findings?
  In this mixed-methods feasibility study, the 12-week personalised exercise therapy and self-management support programme and study methods was found feasible in people with multimorbidity in terms of recruitment rate, retention at follow-up, outcome burden and SAEs, while compliance and the associated low number with a clinically relevant improvement in quality of life and function indicated that some amendments were needed before proceeding to the RCT. The intervention was found acceptable by both participants and physiotherapists with some barriers to participation among participants relating to managing multiple chronic conditions while caring for others or maintaining a job.
- What are the implications of the feasibility findings for the design of the main study?
  To increase the potential effect of the MOBILIZE intervention and based on the qualitative findings, the number of supervised exercise sessions was changed to 24 instead of 18 and the home-based exercise sessions was removed. From the qualitative evaluation, it also became clear that a more thorough training of the facilitators was important, and that special attention and support would be needed for facilitators with less clinical experience. Finally, the 6 90-min self-management support sessions were changed to 24 30-min sessions immediately before the 24 exercise sessions. This was done to adhere to the qualitative findings and to better integrate the two interventions and support long-term adherence by including more topics found relevant by the participants.

## Introduction

Multimorbidity, commonly defined as two or more long-term conditions in the same individual, affects about a third of the world’s population [1] and is considered the next major health priority [2]. Multimorbidity is distinctly different from comorbidity, as it does not give priority to one condition over another leading to patient-centred care instead of disease-centred [3]. Individuals with multimorbidity account for 78% of all consultations in primary care [4] and are more likely to die prematurely, to be admitted to hospital and have an increased length of stay as compared to those with only one condition [5, 6]. Furthermore, multimorbidity is associated with poorer function and quality of life, depression, intake of multiple drugs and increased health care utilisation [7–13], with some studies demonstrating an almost exponential relationship between the number of conditions and their associated costs [11]. In the most deprived areas, onset of multimorbidity occurs 10 years earlier and the prevalence as well as burden is higher as compared to the least deprived areas [3, 14]. With an ageing population, and the fact that most individuals with multimorbidity are younger than 65 years of age [14], the proportion of people with multimorbidity is expected to increase rapidly in the future [15, 16], highlighting the need to take action to address the increasing burden of multimorbidity through treatment and prevention [3].

The most recent systematic review of primary care interventions for individuals with multimorbidity found small to negligible effects on quality of life and function from available interventions and highlighted the need for further high-quality trials [17]. Furthermore, the authors suggested that future research should focus on targeting health behaviours, including physical activity and exercise therapy [17]. While physical activity is any movement that increases energy expenditure, exercise therapy is a specific type of physical activity designed and delivered with specific therapeutic goals, i.e., related to symptoms and function [18]. Systematic reviews investigating the effect of exercise therapy in the most prevalent and disabling single long-term conditions have demonstrated the safety and physical and mental benefits of exercise therapy [19–23]. Furthermore, a recent systematic review demonstrated that exercise therapy was safe and effective also in people with comorbidities [24]. In fact, research on exercise therapy is among the top 10 research priorities for people with multimorbidity [25]. However, no full-scale randomised, controlled trial (RCT) has investigated the effects of exercise therapy in people with multimorbidity [24]. Given the profound impacts of multimorbidity on the individual and the change of behaviour needed in order to integrate physical activity and exercise therapy in daily life, an effective intervention for multimorbidity would also need to integrate self-management support [17, 26]. Evidence on such interventions is important in supporting evidence-based recommendations in clinical practice and could potentially help improve the physical and psychosocial health of individuals with multimorbidity and thereby have a significant societal impact.

In preparation for a full-scale RCT, this mixed-methods study aimed to investigate the feasibility of a 12-week supervised exercise therapy and self-management support programme in people with multimorbidity. We combined quantitative research progression criteria and qualitative interviews focusing on acceptability as this is crucial in uncovering potential issues related to compliance, recruitment, retention, and delivery of the intervention.

## Methods

### Design

This mixed-methods feasibility study was designed to evaluate research progression criteria in preparation for a full-scale definitive RCT, at the same time incorporating qualitative feedback from participants and physiotherapists facilitating the intervention. The study did not include a control group, and therefore there was no blinding of patients, facilitators, or outcome assessors.

The study was reported according to the CONSORT statement extension to randomised pilot and feasibility trials [27]. Furthermore, it adhered to the Declaration of Helsinki, was approved by the Ethics committee (SJ-857) and the data protection office in Region Zealand (REG-015-2020) and University of Southern Denmark (10.918) and pre-registered with the full-scale RCT on ClinicalTrials.gov (NCT04645732).

The feasibility study is part of the MOBILIZE project funded by the European Union’s Horizon 2020 research and innovation programme (grant agreement No. 801790) and comprises all four phases in the Medical Research Council (MRC) framework for developing and evaluating complex interventions [28]. Prior to planning the feasibility study and the RCT, several reviews on exercise therapy [24], behavioural interventions and behaviour change techniques (Jäger M, Zangger G, Bricca A, Dideriksen M, Smith SM, Midtgaard J, Taylor R, Skou ST: Mapping interventional components and behaviour change techniques used to promote self-management in people with multimorbidity: a scoping review, in review) [29], available apps for people with multimorbidity and recruitment and retention [30], cohort studies [31, 32] were done. Furthermore, interviews with people with multimorbidity and their carers, health care providers and patient organisations [33, 34] were conducted to inform the development of the intervention and study design. For more details on the MOBILIZE study, see www.mobilize-project.dk and the Open Science Framework website (https://osf.io/qk6yg/).

### Participants

Adult patients (18 years or older) with multimorbidity fulfilling the eligibility criteria below were included in the feasibility study from the Department of Endocrinology and the Department of Cardiology at Slagelse Hospital, the Department of Pulmonology and the Department of Orthopedics at Næstved Hospital, the Psychiatric Hospital West, Slagelse and posters and social media advertisements.

Inclusion criteria were

- At least two of the following conditions: osteoarthritis (OA), type 2 diabetes (T2D), depression, heart disease (heart failure (HF) or ischemic heart disease (IHD)), hypertension, and chronic obstructive pulmonary disease (COPD). Having other conditions did not exclude a patient.
- Able to walk 3 m without any assistance. A score of 3 or above on the Bayliss Disease Burden Impact Scale [35, 36] for at least one of the conditions listed above and a score of 2 or above for at least one of the other listed conditions. The scale evaluates how much the condition limits daily activities from 1 (not at all) to 5 (a lot) [35, 36].
- Willingness and ability to participate in a 12-week supervised exercise therapy and self-management programme twice a week. Ability could relate to, e.g. transportation options and availability during the 12 weeks, but not to physical capacity to exercise.

Exclusion criteria were

- Participation in supervised systematic exercise for one of their diseases within the last 3 months
- Patients with an unstable health condition or at risk of serious adverse events as evaluated by a medical specialist
- Patients with terminal conditions or with life expectancy of less than 12 months
- Patients categorised as Class IV on the New York Heart Association (NYHA) Functional Classification scale
- Patients with psychosis disorders, post-traumatic stress disorder, obsessive compulsive disorder, attention deficit hyperactivity disorder, autism, anorexia nervosa/bulimia nervosa and patients with an abuse
- Other reasons for exclusion (unable to understand Danish, mentally unable to participate)

### Intervention

Participants underwent the 12-week personalised, supervised exercise therapy and self-management support programme developed based on the previous phases of the MOBILIZE project. For further details on the development process, please refer to the intervention development paper [37]. The intervention was facilitated by physiotherapists trained in study procedures at either Slagelse or Næstved Hospital, Denmark.

The participants were instructed to continue their current treatment or services as provided by their general practitioner or specialist, including medication intake, if needed.

In connection with recruitment, participants received a telephone call from a member of the project team to discuss any barriers or special requirements to personalise the treatment as much as possible. Based on prior trial experience [38], this had the potential to support retention of the participants in the study. The programme consisted of an individual introduction with a physiotherapist to set goals and determine exercise level and 18 supervised exercise sessions, six home-based exercise sessions and six self-management sessions distributed across the programme. Details of the programme are reported according to the Template for Intervention Description and Replication (TIDieR) [39] in Table 1.

**Table 1:**
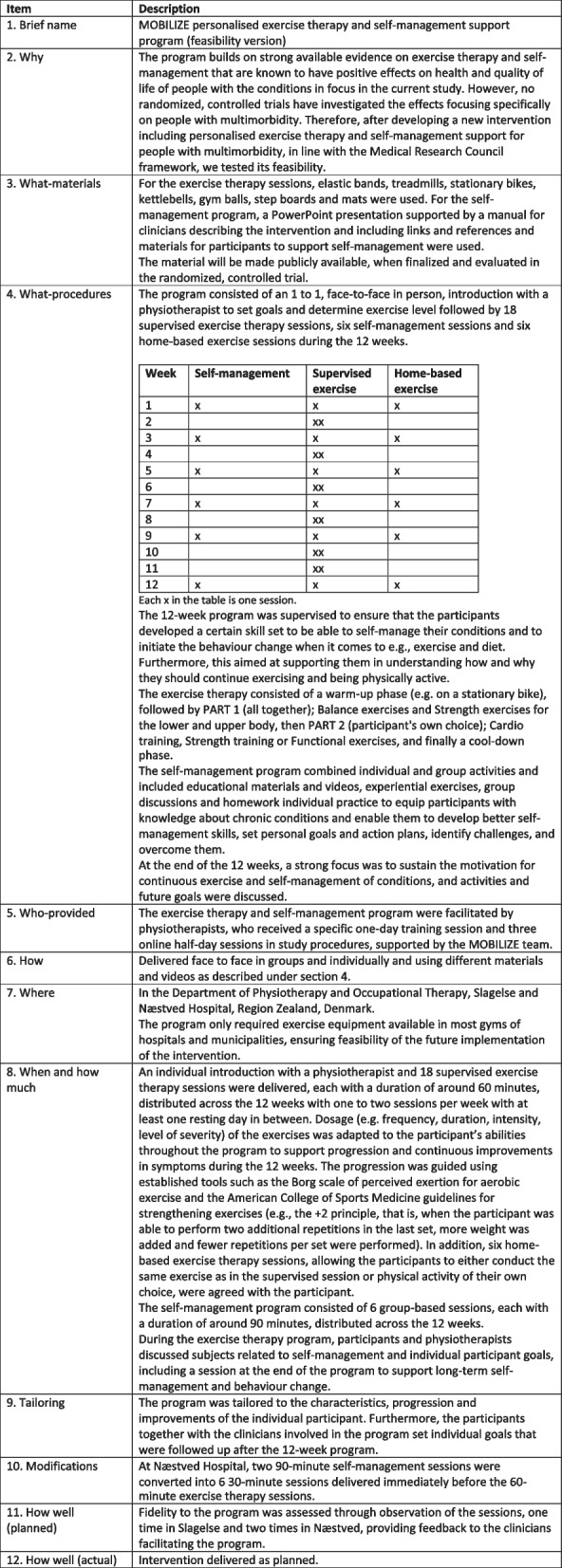
Details of the exercise therapy and self-management programme described according to the Template for Intervention Description and Replication (TIDieR) [39]

### Feasibility outcomes

The predefined progression criteria of the feasibility study followed a red/amber/green traffic light system, which is preferred instead of a simple stop/go basis [40]. The progression criteria are presented in Table 2. Together with the findings from the qualitative interviews with participants and physiotherapists from the feasibility study, the results of the progression criteria were evaluated by the MOBILIZE team to decide whether to proceed with the RCT or which amendments of the intervention or study design that were needed to proceed. The scientific advisory board and other relevant stakeholders could be consulted, if needed.

**Table 2:** Progression criteria to proceed with the randomised controlled trial

Proceed with RCT	Proceed, but changes to the protocol need to be discussed	Do not proceed with main trial unless the problem can be solved
Recruitment of 20 participants with multimorbidity within 3 months	Recruitment of 20 participants with multimorbidity within 3–6 months	20 participants with multimorbidity are not recruited within 6 months
At least 75% retention of participants through follow-up	At least 50% retention of participants through follow-up	Less than 50% retention of participants through follow-up
At least 75% complete more than half of the supervised exercise therapy sessions and half of the self-management sessions	At least 50% complete more than half of the supervised exercise therapy sessions and half of the self-management sessions	Less than 50% complete more than half of the supervised exercise therapy sessions and half of the self-management sessions
At least 80% of participants do not find the outcome assessment so burdensome that they would not participate in the study again	At least 70% of participants do not find the outcome assessment so burdensome that they would not participate in the study again	Less than 70% of participants do not find the outcome assessment so burdensome that they would not participate in the study again
Improvements in quality of life and function found clinically relevant by at least 50% of the participants	Improvements in quality of life and function found clinically relevant by at least 25% of the participants	Improvements in quality of life and function found clinically relevant by less than 25% of the participants
No serious intervention-related adverse events during follow up	Less than five serious intervention-related adverse events during follow up	Five or more serious intervention-related adverse events during follow up

Burden of outcome assessment was evaluated by two yes/no questions asking the participants if they found the tests and assessments (conducted at the study site) or questionnaires (responded to via an online link or paper at home) so burdensome that they would not participate in the study again. Both questions were asked as the last questions of the questionnaire after completing the baseline tests and assessments. Health-related quality of life was evaluated as change in the descriptive index of the EQ-5D-5L (− 0.624 to 1; worst to best) from baseline to the 3-month follow-up (i.e. immediately after the exercise therapy and self-management programme). We also evaluated the participants’ self-rated health on the EQ visual analogue scale (EQ VAS, 0 to 100, worst to best) [41, 42]. The descriptive index of the EQ-5D-5L questionnaire (converted from a string of five integers to an index score using time-trade-off-based weights from the Danish crosswalk value set [43] is a reliable and valid measure of general health/quality of life [41, 42], and a change of 0.074 is considered clinically relevant in people with different long-term conditions based on a UK-based study [44].

Function was evaluated as change in distance walked during the 6-min walk test (6MWT) from baseline to the 3-month follow-up. The 6MWT is a reliable and valid measure of physical function that is widely used in people with long-term conditions and multimorbidity [24, 45], and a change of 30.5 m is considered clinically relevant in people with different long-term conditions [46].

Adverse events (AE) and serious adverse events (SAE) were recorded at the 3-month follow-up by asking the participants about potential AEs using open-probe questioning to ensure that all AEs were recorded. Furthermore, the medical records of the patients were checked for all AEs occurring from inclusion until the 3-month follow-up. AEs were classified according to the Food and Drug Administration definition of an SAE [47].

High compliance was defined as attending at least 14 out of 18 sessions and four out of the six self-management sessions.

### Qualitative evaluation

The aim of this qualitative part was to investigate the participants’ and facilitators’ experiences of taking part (or delivering) the exercise and self-management intervention and to explore their perceptions of the intervention’s acceptability. This is in line with the MRC Framework [28] that stresses the importance of assessing the perceptions, needs, capabilities and experiences of intervention recipients as well as those delivering the intervention prior to embarking on a full-scale RCT. This helps maximise value by ensuring that the intervention is optimised at the trial stage. We conducted semi-structured telephone interviews with both participants and facilitators in Danish. The interviews were audio-recorded and transcribed verbatim and translated into English.

### Sample size

We did not aim for statistical power to be able to identify quality of life and functional improvements but recruited 20 participants to ensure that a sufficient number completed the follow-up to allow us to meaningfully interpret progression criteria. Generally, 12 participants are considered a rule of thumb for pilot and feasibility studies from a feasibility, regulatory and statistical perspective [48]. With a sample of 20, the widest 95% confidence interval would be found with a 50% proportion (10/20), ranging from 27 to 73%.

### Data analyses

Except for recruitment, feasibility outcomes were analysed descriptively as proportions of participants satisfying each criteria with 95% Confidence Intervals (CIs; calculated using the “metaprop”, “exact” option in STATA). Recruitment was summarised as number of days to recruit the 20 participants. Participant characteristics were summarised as mean (standard deviation), median (range) and N (%) as appropriate. All analyses were performed in SPSS (IBM SPSS Statistics 28, Armonk, NY, USA) or STATA (StataCorp, version 17, College Station, TX, USA).

The qualitative analysis included an iterative hybrid approach based on inductively exploring the participants’ and facilitators’ experiences while deductively applying the Theoretical Framework of Acceptability (TFA) [49] to the data to investigate acceptability. The analysis followed the six steps of Thematic analysis [50]. Two researchers (RB and MJ) collaborated closely in the process of performing the analysis. First, they read the transcripts and notes several times to gain familiarity, followed by identifying codes in the data that were subsequently grouped into themes, in an iterative fashion. Coding was performed in NVIVO 12 (Vivo qualitative data analysis software; QSR International Pty Ltd. Version 12, 2018) by assigning labels to excerpts of the data to get an overview. The two researchers developed themes and subthemes by collating similar codes and then subsequently refined them. They discussed and reviewed the initial themes and moved back and forth through the data to ensure coherence and reflexivity. After several iterations, a consensus had been reached on the final version of the themes and subthemes, and quotes were extracted to support the themes and ensure transparency and consistency. The final step was producing a narrative analysis including an interpretation of the findings and supporting quotes.

### Patient and public involvement

The MOBILIZE project is committed to patient involvement and has included patients living with multimorbidity and their carers in all aspects of the decision-making process in the project (collaborate level on the IAP2 Spectrum of Public Participation [51]). Their experiences, needs, and preferences played an important role in the development of the intervention, study design and conduct as well as evaluation on how to adapt the intervention and study design following the feasibility study.

## Results

### Patient characteristics

**Fig. 1:**
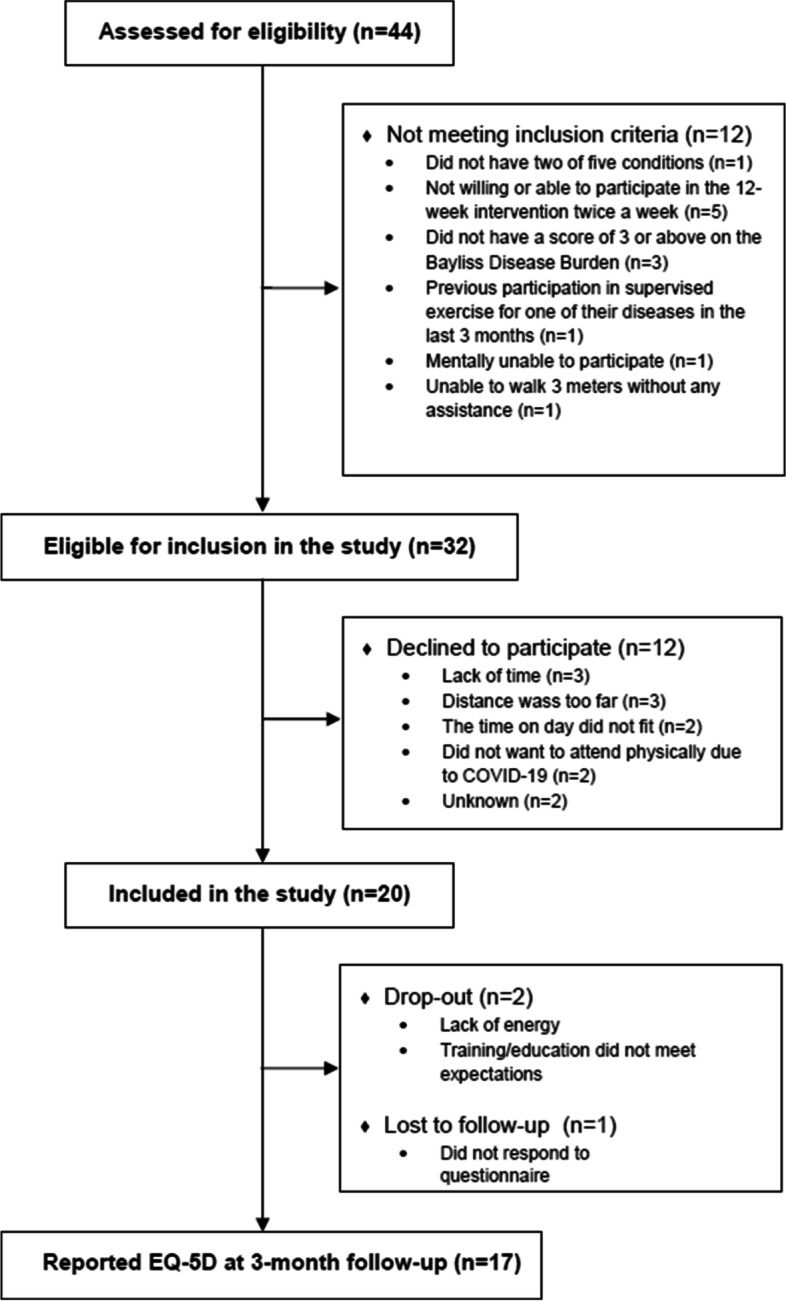
Study flow

Forty-four patients were assessed for eligibility between February 22 and April 12, 2021 (Fig. 1), and 20 participants (8 females; mean age (SD) 67 (6.9), corresponding to 45% of the population assessed for eligibility and 63% of those eligible for inclusion) were included (Table 3), out of which eight had participated in a previous qualitative focus-group interview study [34]. The most prevalent long-term conditions were hypertension (*n* = 14) and knee or hip OA (*n* = 12), followed by COPD (*n* = 9), T2DM (*n* = 8), heart disease (*n* = 6) and depression (*n* = 5), also reflected in the most prevalent combination of conditions which were hypertension and OA (*n* = 9), hypertension and COPD (*n* = 7) and hypertension and T2DM (*n* = 7). One person with well-controlled post-traumatic stress disorder was included, despite of it being an exclusion criterion. The person completed the intervention as the rest of the participants.

**Table 3:** Baseline characteristics^a^

Characteristic	
Age, mean (SD) years	67.1 (6.9)
Females, *N* (%)	8 (40%)
Educational level, short-term education or lower, *N* (%)	14 (70%)
Body mass index, mean (SD)	32.0 (7.2)
Number of long-term conditions, mean (SD), median (range)	9.2 (4.0), 10 (3–19)
Bayliss Burden of Illness Measure, mean (SD), median (range)	21.0 (10.8), 19 (9–48)
Multimorbidity Treatment Burden Questionnaire^a^, mean (SD), median (range)	13.9 (10.6), 12.5 (2.5–35.0)
12-item WHO Disability Assessment Schedule 2.0^b^, mean (SD), median (range)	22.8 (13.1), 22.9 (2.1–50.0)
EQ-5D-5L Index^c^, mean (SD)	0.65 (0.13)
EQ-5D VAS^d^, mean (SD)	46.2 (19.6)
6-min walk test, mean (SD) meters	380.9 (122.7)

Missing values (numbers): Education level (1 missing); Number of long-term conditions (1); Bayliss Burden of Illness Measure (1); multimorbidity Treatment Burden Questionnaire (1); 12-item WHO Disability Assessment Schedule 2.0 (1); EQ-5D-5L Index (1); EQ-5D VAS (1); 6-min walk test (2)

^a^Multimorbidity Treatment Burden Questionnaire: (0–100; no burden (score 0), low burden (score < 10), medium burden (10–22), high burden (>= 22)

^b^12-item WHO Disability Assessment Schedule 2.0: (0–100; higher scores indicating higher disability)

^c^EQ-5D-5L Index: *(-0.624 to 1; worst to best)*

^d^EQ-5D VAS: (0-100; a low score indicating a bad health state)

### Progression criteria

According to the pre-defined progression criteria, recruitment rate, retention, outcome burden and SAEs were acceptable in terms of proceeding with the RCT. On the other hand, compliance and improvements indicated that amendments were needed before proceeding to RCT (Table 4).

**Table 4:** Progression criteria results

Proceed with RCT	Proceed, but changes to the protocol need to be discussed	Do not proceed with main trial unless the problem can be solved
20 participants recruited in 49 days		
17 out of 20 (85%, 95% CI 62% to 97%) completed the 3-month follow-up		
	14 out of 20 (70%, 95% CI 46% to 88%) completed at least half of the exercise sessions and 70% (95% CI 46% to 88%) at least half of the self-management support sessions	
16 out of 17 participants (95%, 95%CI 71 to 100%) did not find the questionnaires to burdensome and 100% the assessments		
	7 out of 20 participants (35%, 95% CI 15 to 59%) had clinically relevant improvements in quality of life, and 9 out of 20 participants (46%, 95% CI 23 to 68%) in function	
No serious intervention-related adverse events were identified		

Compliance (mean (SD, range) sessions completed) were 11.7 (5.3, 0–17) out of 18 and 3.8 (1.8, 0–6) out of 6 for the supervised exercise and self-management support sessions, respectively. Ten (50%) and 14 (70%) had high compliance with the supervised exercise and self-management support sessions, respectively, according to the pre-defined criteria.

Six people experienced eight AEs during the 3 months of follow-up (Table 5).

**Table 5:** Adverse events

Adverse event	Consultation or treatment	Duration	Related to study intervention	Serious adverse event	Consequence for study participation
Cystitis^a^	Admitted to the hospital	Less than a week	No	Yes	Stopped, unclear if it was due to the adverse event
Back pain^a^	Unknown	1–2 weeks	Unclear	No	Stopped, unclear if it was due to the adverse event
Chest pain	Emergency room that did not find any irregularities	45 min	No	No	None
Issues with a scar after tendon surgery in hand	Unknown	Unknown	No	No	None
Minor ankle sprain at home	Emergency room and general practitioner	Unknown	No	No	Unclear, but did not stop
Depression relapse^b^	Admitted to the psychiatric hospital	Unknown	No	Yes	Unclear, but did not stop
Leg pain^b^	No findings on X-ray	Short-lasting	No	No	Unclear, but did not stop
Chest pain	Emergency room that considered it to be muscle pain after manual labour	4 days	No	No	None

^a^The same participant had these two adverse events

^b^The same participant had these two adverse events

### Qualitative evaluation results

We conducted the interviews in April 2021. The respondents had undergone between eight and eleven of the 24 exercise sessions and two of the six self-management sessions at the time of the interviews. All interviews lasted between 30 and 75 min.

Eleven participants (6 females, mean (SD) age 65.3 (6.3) years) and 2 facilitators (both females, with 3 and 29 years of experience as a physiotherapist) participated in the qualitative interviews. Most of the participants in the interviews were retired (9 out of 11, while only one was working and the other volunteering). They experienced a combination of mental, rheumatic, musculoskeletal, pulmonary, cardiovascular, endocrine, gastrointestinal, and neurological conditions and had participated in between 3 and 8 of the intervention sessions at the time of interview.

Based on the interviews, we identified four major themes through thematic analysis: (1) the value of an individualised and holistic approach to multimorbidity; (2) ‘we are a bag of mixed sweets’*—*the social benefits of being in a group; (3) the importance of exercise motivation; and (4) burden of living with multiple conditions and contextual-related challenges. See Additional file 1 for a selected list of quotes within each theme.

#### Theme 1: the value of an individualised and holistic approach to multimorbidity

This theme captures the participants’ positive attitudes towards the programme and their acknowledgement that MOBILIZE was holistic, as it addressed all their conditions and limitations as opposed to one condition at a time.

“I think it’s good that somebody sat down and thought ‘now we’ll try to develop a programme and see if it can do any good for some of these old folks who are in pain’. Just coming up with that idea, I think it’s great.” (Participant B)

They also shared their appreciation for the individualised and supervised nature of the exercise sessions, which appeared to increase the feeling of safety and being supported while exercising.

“And then there is of course the physiotherapist. Having a physiotherapist to assist you, well that’s worth a lot!” (Participant B)

Furthermore, participants expressed that the self-management sessions were useful, relevant, and provided them tools to manage their long-term conditions, despite some initial scepticism and some minor critiques of their content and structure (e.g. length).

“At first I thought, ‘why do we have to do all six (sessions).’ It must be possible to do it in less. But after we had these first two, I’m thinking ‘oh, ok now I can see why.” (Participant D)

Facilitating an individualised and holistic programme was also perceived positively by the physiotherapists, but there was a caveat. For one of them, who had less clinical experience, facilitating the self-management sessions appeared to be challenging.

“As a physiotherapist, it can be a bit overwhelming and a bit difficult to stand there and talk about how to handle depression and anxiety symptoms or talk about diets or mindfulness. Things I am not trained to do.” (Facilitator B)

This highlights a need to offer more training and better support to physiotherapists when they facilitate sessions focusing on mental health or healthy dietary habits.

#### Theme 2: ‘we are a bag of mixed sweets’—the social benefits of being in a group

Being part of a group was one of the most valued aspects of the programme and appeared to yield enjoyment while also improving motivation and adherence to the programme. Furthermore, exercising with a group in a structured manner was something that contributed to a sense of accountability and increased motivation. Some highlighted that participating in a group programme alongside people who experienced similar issues seemed valuable, while others acknowledged that the group was indeed mixed, in terms of managing different combinations of conditions, but that despite of that many of their problems were the same.

“We are a bag of mixed sweets. (…) Someone like me has these problems, and others they really do have some serious problems with COPD, for example, right. And some have problems with diabetes. (…) Our needs are different. (…) but when you go down to the roots of it, then many of the problems are the same.” (Participant E)

Finally, both the participants and facilitators agreed that smaller groups are preferable because they are easier to facilitate, better in terms of facilitating individualised supervision and better rapports within the group.

“As a physiotherapist, I like to pay close attention to some of the patients quite a lot. So, I think ten would be too many.” (Facilitator B)

#### Theme 3: the importance of exercise motivation

This theme reflects a mixed picture, where some participants expressed that they felt safe exercising without supervision and that they had no issues with the home-based programme while others shared that they did not perform the exercises, mainly due to lack of time or low motivation.

“I do not get [the home-exercise] done. Well, there is so much else I need to do. Well, I have a house and a garden, and it must be kept, and – yes.” (Participant J)

Moreover, one facilitator acknowledged that for one of the participants it might have been too burdensome to engage in the home-based programme in addition to the core programme. This was due to her being a carer for her husband and having household responsibilities as well.

“She has a lot on her plate, and she is physically active at home, keeping a house and a garden. And she can’t manage any more. Just being here, with all the things she has and a sick husband, that is an accomplishment in itself. So I can’t pressure her to do more.” (Facilitator A)

#### Theme 4: burden of living with multiple conditions and contextual-related challenges

This theme captures some of the difficulties experienced by the participants during the programme. These were related to functional limitations (poor balance, symptom exacerbation) that hindered participation. Moreover, despite that many of the participants were retired, they were not only caring for themselves but also for their spouses, supporting them to navigate the system and manage their conditions. This appeared to be prioritised over attending the sessions. For one of the few participants that were not retired, the difficulty arose from having to leave his work early to arrive on time for the exercise session. He shared that this created frustration and guilt, making him doubt that he was able to continue taking part.

“And then you drive off from your co-worker and say, ‘hurry up and finish’. I’ll just take off. I have to go exercise.” (Participant D)

In addition to these challenges, several of the participants highlighted some contextual-related issues (lack of parking, needing a larger room).

## Discussion

A 12-week personalised exercise therapy and self-management support programme was found feasible in people with multimorbidity in terms of recruitment rate, retention at follow-up, outcome burden and SAEs, while compliance and the associated lack of improvements in quality of life and function indicated that some amendments were needed before proceeding to the RCT. The intervention was found acceptable by both participants and physiotherapists with some barriers among participants relating to managing multiple chronic conditions while caring for others or maintaining a job. Physiotherapists expressed a need for additional training, to be better equipped in delivering the self-management sessions particularly.

We pre-defined the progression criteria and their evaluation based on existing recommendations for feasibility studies [40, 52]. We used a traffic light system, and no progression criterion was red, i.e. “*do not proceed with the trial”*, and the criteria that needed attention (compliance and improvements in quality of life and function) were close to being acceptable (i.e. green). Supported by the mostly positive feedback from participants and facilitators, this highlights that the intervention was feasible. On the other hand, the study also highlighted some areas that need to be amended to proceed with the RCT with confidence that the intervention will have superior effect on quality of life and function as compared to usual care alone. Our feasibility study was not powered to study the effect size of the intervention [52]. Yet we pay attention to the relatively low number of people experiencing a clinically relevant improvement, which unfortunately is in line with findings in the most recent systematic review in the field demonstrating only small to negligible effects on quality of life and function from available interventions [17]. One of the largest high-quality RCTs on multimorbidity so far, the 3D-trial [53], investigated the effects of one 6-monthly comprehensive multidisciplinary patient-centred review in general practice as compared to usual care. Their main outcome was EQ-5D-5L, and while they did not find any difference between groups at 15 months (mean (95% CI) 0.00 (− 0.02 to 0.02)), both groups had an approx. 0.04 lower EQ-5D-5L score at follow-up as compared to baseline [53]. This suggests that preserving the same level of quality of life (and function) over time in people with multimorbidity, instead of improving, could be a more realistic and appropriate goal and lends support to proceeding with the RCT of our intervention. To increase the potential effects of the MOBILIZE intervention, and based on the findings of this feasibility study, including the qualitative findings, we decided to increase the number of supervised exercise sessions to 24 instead of 18 and to remove the home-based exercise sessions. Hopefully, this will help increase the compliance with the sessions and thereby the effect, without compromising the long-term integration of physical activity and exercise in daily life. To mitigate the risk of reduced adherence over time, the self-management support programme has an integrated component aimed at supporting long-term self-management and behaviour change.

From the qualitative evaluation, it also became clear that a more thorough training of the facilitators was important, and that special attention and support would be needed for facilitators with less clinical experience. Interestingly, one of the challenges we had expected, that the heterogeneity of people with multimorbidity [54] would make it impossible to include them all in the same group, was not experienced by all but one participant. In fact, they highlighted that they had many common challenges in their daily life. This is encouraging and supports providing the recommended person-centred care, e.g. focusing on improving function, instead of disease-centred care and rehabilitation [26, 55]. Finally, the 6 90-min self-management support sessions were changed to 24 30-min sessions immediately before the 24 exercise sessions to adhere to the qualitative findings and to better integrate the two interventions and support long-term adherence by including more topics found relevant by the participants. For further details on the development process and changes to the intervention please refer to Bricca et al. [37].

To the authors’ knowledge, no prior high-quality RCTs have investigated the effects of personalised and supervised exercise therapy and self-management support targeting people with multimorbidity specifically [17], despite the promising effects demonstrated in RCTs of people with long-term conditions and comorbidities [24]. Four pilot RCTs of supervised exercise therapy have shown mixed results in terms of improvements in physical function [56–59], highlighting the need for further fully powered, definitive RCTs. The MOBILIZE RCT will help fill that gap and provide the first evidence on the effects of supervised exercise therapy and self-management support that can hopefully help the growing number of people worldwide affected by multimorbidity. Additionally, it will be important to see whether the participants in the RCT are able to maintain a physically active lifestyle in the long term, due to the significant impact of physical activity on overall health, prevention of long-term conditions and death [60–62].

### Strengths and limitations

The strengths of the study include the thorough preparation and development phase of the study design and intervention as well as the involvement of patients, carers, multidisciplinary clinicians and other stakeholders throughout the MOBILIZE project. This supports the feasibility and clinical relevance of the intervention as well as the potential subsequent implementation. Furthermore, by combining quantitative and qualitative methodologies we gained a more comprehensive and clinically useful understanding of the intervention’s feasibility. The inherent methodological limitations of a one-armed feasibility study, including small sample size, lack of control group and blinding of participants and the researchers, precludes any conclusions on the effectiveness of the intervention. By limiting our population to specific conditions and excluding, e.g. people with a short life expectancy, conclusions from the feasibility study as well as the subsequent RCT cannot be generalised to all people with multimorbidity. However, targeting people with specific combination of conditions, e.g. linked by physiological factors (systemic inflammation) and risk factors (physical activity) as done in our study, is recommended as a way to deal with the complexity of managing multimorbidity [54], and our intervention follows the recommended patient-centred approach to care, not giving priority to one condition over another [3]. Furthermore, we did not exclude patients with certain conditions from the study.

## Conclusions

A 12-week personalised and supervised exercise therapy and self-management support programme is feasible in people with multimorbidity. The subsequent RCT, amending the intervention according to progression criteria and feedback emerging from this feasibility study, will determine whether the programme is superior to usual care alone in improving quality of life and function.

## Supplementary Information

**Additional file 1.** Quotes from respondents within each of the four major themes identified in the thematic analyses.

## Data Availability

Any data not represented in the manuscript or additional file is available from the first author upon reasonable request.
