# Supplementary material for: Personalised exercise therapy and self-management support for people with multimorbidity: feasibility of the MOBILIZE intervention
Source: Pilot Feasibility Stud. 2023 Jan 18;9:12. doi: 10.1186/s40814-023-01242-0 (PMC9847074; doi:10.1186/s40814-023-01242-0)
Supplement: Supplementary file 1 — Additional file 1. Quotes from respondents within each of the four major themes identified in the thematic analyses. [file 40814_2023_1242_MOESM1_ESM.pdf]

Additional file 1: Quotes from respondents within each of the four major themes identified in the thematic analyses

**The value of an individualized and holistic approach to multimorbidity**	**We are a bag of mixed sweets'- the social benefits of being in a group**	**The importance of exercise motivation**	**Burden of living with multiple conditions and contextual-related challenges**
'*At first I thought, 'why do we have to do all six (sessions).' It must be possible to do it in less. But after we had these first two ones I'm thinking 'oh, ok now I can see why.'* (Participant D)	*'I am one of those people who don't mind working out alone. I am able to do that. But it is significantly more fun when there are others. (…) The group dynamic does something, and there are some who really need the group dynamic to be there, right?'* (Participant E)	*'Well, because then, there is no one there, you walk around alone, right? Then you think 'oh yes, well I do the exercises' and 'well you know what, you might as well stop now, right? But you must just do 20 more, right? That is why you want someone to be there. When you do not have someone who can hold you to it, so to speak, right?* (Participant C)	*'And then you drive off from your co-worker and say, 'hurry up and finish'. I'll just take off. I have to go exercise.'* (Participant D)
'*Because she is - she knows a whole lot about physical therapy, but she doesn't know much about the psychological stuff. (…) Now that you mention it, we focused on diet at some point. But instead of a nutritionist, who knows a lot about diet, she was trying to read through these slides- and it has nothing to do with her, so it is not criticism at all, but my expectations were certainly not fulfilled in that area.'* (Participant H)	*'And then you also get something social from it, right? We have a little fun, right, when we exercise, don't we? And we do get along very well and have a good time together, right?' (Participant C)*	*'She has a lot on her plate, and she is physically active at home, keeping a house and a garden. And she can't manage any more. Just being here, with all the things she has and a sick husband, that is an accomplishment in itself. So I can't pressure her to do more. (Facilitator A)*	*'I'll get sick with stress when I can't find parking and if I have to park far away and so on. Then I thought to myself, 'I can't do this'. It stresses me out so much that I can't do it.* '(Participant I)
As a physiotherapist, it can be a bit overwhelming and a bit difficult to stand there and talk about how to handle depression and anxiety symptoms or talk about diets or	*'So, also, there's sometimes you think when you get up in the morning," no, I don't want to drive to training today', and then you think 'oh yes, of course*	*'And I know it's only three months and then I don't know what will happen. But then I will simply have to pull myself together and find some fitness-*	*'There is only one session that I have missed. And that was, I had a fall that Friday and didn't attend the session the following Monday. You*

*mindfulness. Things I am not trained to do. ‘(*Facilitator B)	*you will do it, because the others expect you to come’*. (Participant A)	*centre appropriate for my age. ‘*(Participant B)	*see, my senses and my stubbornness were fighting, and that time my senses won.’ (Participant G)*
‘*I think it’s good that somebody sat down and thought ‘now we’ll try to develop a program and see if it can do any good for some of these old folks who are in pain’. Just coming up with that idea, I think it’s great.’* (Participant B)	*I think it is nice to meet other people, so that you might say, ‘well you're not the only one who is in your situation, right’. And, to get to know other people, and what they struggle with.’* (Participant A)	*‘And I know it’s only three months and then I don’t know what will happen. But then I will simply have to pull myself together and find some fitness-centre appropriate for my age. ‘*(Participant B)	
‘*I have been admitted many times to the psychiatric department. And there they take care of my psychiatric conditions, and the rest, they couldn’t care less about it to put it mildly. And I would prefer it if they would take care of more conditions at the same time.*’ (Participant G)	*‘As a physiotherapist, it feels like you have to pay close attention to some of the patients quite a lot. […] So I think 10 would be too many.’* (Facilitator B)		
‘And then there is of course the physiotherapist. Having a physiotherapist to assist you, well that’s worth a lot!’ (Participant B)			
